# Insights into open/closed conformations of the catalytically active human guanylate kinase as investigated by small-angle X-ray scattering

**DOI:** 10.1007/s00249-015-1079-9

**Published:** 2015-10-07

**Authors:** Rohit Jain, Nazimuddin Khan, Andreas Menzel, Ivan Rajkovic, Manfred Konrad, Simone Techert

**Affiliations:** Max Planck Institute for Biophysical Chemistry, Am Fassberg 11, 37077 Göttingen, Germany; Paul Scherrer Institute, 5232 Villingen, Switzerland; Institute for X-ray Physics at University of Göttingen, Friedrich-Hund-Platz 1, 37077 Göttingen, Germany; FS-SCS at DESY, Notkestraße 85, 22607 Hamburg, Germany

**Keywords:** Protein conformations, Enzyme, Guanylate kinase (GMPK), Nucleotide kinase, Small-angle X-ray scattering (SAXS)

## Abstract

**Abstract:**

Bio-catalysis is the outcome of a subtle interplay between internal motions in enzymes and chemical kinetics. Small-angle X-ray scattering (SAXS) investigation of an enzyme’s internal motions during catalysis offers an integral view of the protein’s structural plasticity, dynamics, and function, which is useful for understanding allosteric effects and developing novel medicines. Guanylate kinase (GMPK) is an essential enzyme involved in the guanine nucleotide metabolism of unicellular and multicellular organisms. It is also required for the intracellular activation of numerous antiviral and anticancer purine nucleoside analog prodrugs. Catalytically active recombinant human GMPK (hGMPK) was purified for the first time and changes in the size and shape of open/closed hGMPK were tracked by SAXS. The binding of substrates (GMP + AMPPNP or Ap5G or GMP + ADP) resulted in the compaction of size and shape of hGMPK. The structural changes between open and completely closed hGMPK conformation were confirmed by observing differences in the hGMPK secondary structures with circular dichroism spectroscopy.

**Graphical abstract:**

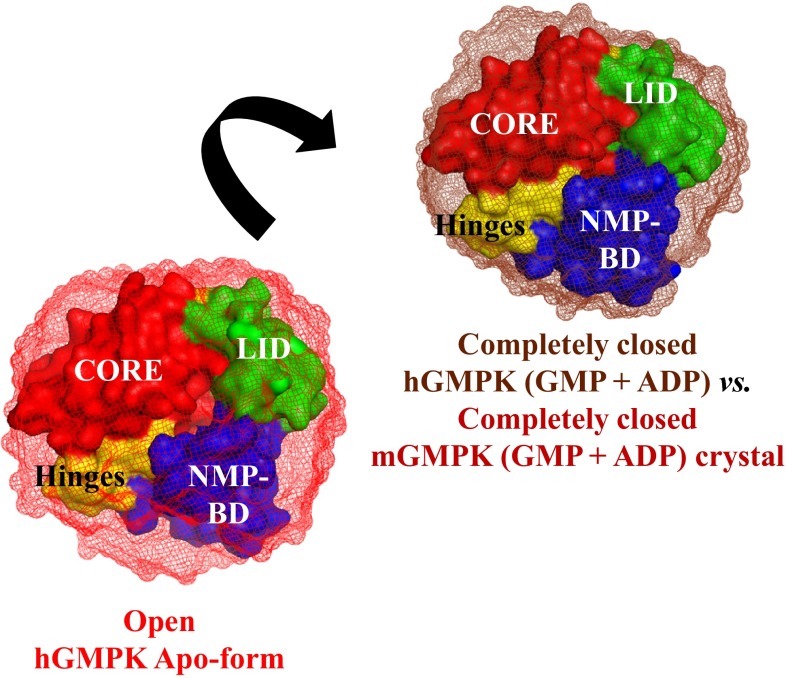

**Electronic supplementary material:**

The online version of this article (doi:10.1007/s00249-015-1079-9) contains supplementary material, which is available to authorized users.

## Introduction

Guanylate kinase (GMPK, ATP:GMP phosphotransferase, EC 2.7.4.8) is an essential enzyme involved in the guanine nucleotide metabolism of unicellular and multicellular organisms. It catalyzes the reversible phosphoryl group transfer from ATP to GMP yielding GDP and ADP (Beck et al. 2003; Choi and Zocchi 2007; Sekulic et al. 2002). GMPK plays an important role in the recycling of the secondary messenger cGMP and thereby regulates the supply of guanine nucleotides to various signal transduction pathways (Hall and Kuhn 1986; Konrad 1992). In addition to its physiological roles, GMPK is also required for the intracellular activation of numerous antiviral and anticancer purine nucleoside analog prodrugs (Sekulic et al. 2002). Prominent examples are 6-thioguanine and 6-mercaptopurine, which are used for the treatment of acute lymphoblastic leukemia and as immunosuppressive agents, respectively (Ardiani et al. 2009; Karran 2006; Karran and Attard 2008; Zhang et al. 2013). Moreover, efficient agents routinely used for the treatment of herpes infections, such as ganciclovir and acyclovir, are first phosphorylated by a viral kinase and then converted to the diphosphate forms by cellular GMPK (Sekulic et al. 2002).

**Fig. 1:**
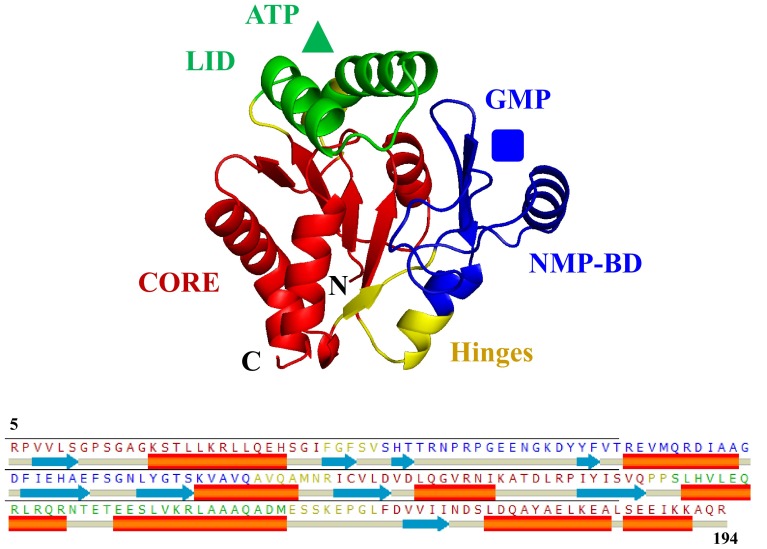
The Swiss homology model for hGMPK was constructed by using the crystal structure of mGMPK’s closed conformation (residues 5-194 of 197 aa) as template [*pdb 1LVG* (Sekulic et al. 2002)]. The three major structural regions present in hGMPK are designated and color-coded as the NMP-binding domain (NMP-BD) (*blue*), the CORE (*red*), and the LID (*green*). These structural regions in hGMPK (residues 5-194 of 197 aa) are interconnected by four hinges (*yellow*). hGMPK substrates, GMP (*blue square*), which bind to NMP-BD and substrate ATP (*green triangle*), which bind to LID in GMPK are schematically presented

GMPK is a member of the family of ATP:NMP phosphoryltransferases (nucleoside monophosphate kinases; NMP kinases, or NMPKs). NMP kinases of different species (*E. coli*, yeast, mouse, and human) have been characterized for their structure and function showing similarities to the class of nucleoside kinases (e.g., human deoxycytidine kinase). NMP kinases and nucleoside kinases share distinctive structural features: the phosphate-binding loop (P-loop) as a common motif found in most ATP- and GTP-binding proteins, the NMP-binding domain (NMP-BD), a LID domain, and a CORE domain, which are connected by dynamic hinges (Hazra et al. 2010; Lavie et al. 1998a, b; Ostermann et al. 2000) (Fig. 1). NMP kinases are bi-substrate enzymes that bind both substrates simultaneously to catalyze phosphoryl transfer. These kinases exist in different conformations: without ligands (apo-form), NMP-bound form, ATP-bound form, and a ternary complex with bound NMP and ATP (closed form). The binding of substrates to NMP kinases reassembles their active centers resulting in large domain movements (Muller-Dieckmann and Schulz 1995). Large conformational changes in NMP kinases were first observed in adenylate kinases (AMP kinases; AKs) (Gerstein et al. 1993; Schulz et al. 1990). Structural analysis of various homologous forms of AKs showed that both ATP and AMP induce substantial conformational changes upon binding to these enzymes. The binding of AMP resulted in the closure of the NMP-BD domain, whereas the binding of ATP caused the closure of the LID domain (Yan and Tsai 1999). The analysis of 17 crystal structures from the NMP kinase family confirmed the existence of these large conformational changes, which were mainly attributed to rigid-body movements of the LID and NMP-BD with respect to the CORE domain (Vonrhein et al. 1995). Similar observations were made for yeast GMPK (yGMPK). Large movements of the GMP-binding domain and smaller but significant movements of the LID domain were reported by comparing the open yGMPK (apo) conformation and the partially closed yGMPK (GMP) conformation. Also, the open yGMPK conformation was found to be more open than its substrate-bound partially closed conformation (Blaszczyk et al. 2001).

Despite their important catalytic and therapeutic roles, the only structural information available for mammalian GMPKs is the crystal structure of the fully closed form of mouse GMPK in complex with one substrate and one product molecule (mGMPK (GMP + ADP), *pdb 1LVG*) (Sekulic et al. 2002). The lack of structural and dynamical information for hGMPK could be due to the purification of recombinant hGMPK in the inactive form and the failures of crystallization (Ardiani et al. 2009; Brady et al. 1996; Sekulic et al. 2002). Small-angle X-ray scattering (SAXS) only requires small amounts of purified hGMPK in solution to provide global structural information, assess structural changes, and derive the three-dimensional surface reconstructions of hGMPK in the absence and presence of its substrates and analogs (Jacques and Trewhella 2010; Jain et al. 2013; Koch et al. 2003; Mallik et al. 2012; Petri et al. 2011, 2012; Putnam et al. 2007; Solanki et al. 2014; Taylor et al. 2012; Tuukkanen and Svergun 2014). We succeeded in purifying the active recombinant hGMPK, which was used to obtain insights into between open/closed hGMPK conformations. SAXS measurements were performed at a home X-ray source and at a third-generation X-ray synchrotron. In these studies, the open hGMPK conformation (no ligand) was compared with three different completely closed hGMPK conformations [GMP + AMPPNP (1), Ap5G (2), and GMP + ADP (3)]. We also evaluated the reconstructed SAXS models (ab initio) of different hGMPK conformations with the crystal structure of its murine homologue, the fully closed form of mGMPK (Sekulic et al. 2002). Different hGMPK conformations were also investigated with circular dichroism to confirm small structural differences observed with SAXS. These investigations may explain the mode of interaction of anticancer and antiviral therapeutics with hGMPK, which is a critical enzyme for the metabolic activation of certain nucleoside analogs.

## Materials and methods

### Cloning, expression, and purification of human guanylate kinase

The 591-bp open reading frame (ORF) of hGMPK (UniProt entry Q16774, also called GMP kinase, GUK1, or GMK), was amplified via polymerase chain reaction (PCR) using the DNA template reported previously (Kuhlendahl et al. 1998; Kumar et al. 2000; Prinz et al. 1999). The final construct includes an N-terminal hexa-histidine tag followed by the SUMO (small ubiquitin-related modifier; SUMO family protein SMT3 of 101 residues)-tag, which was used to improve heterologous protein solubility and stability (Panavas et al. 2009). The detailed hGMPK cloning and purification procedure is described in the supplementary information. For overproduction of the enzyme, the *E. coli* expression strain BL21-(DE3)-pLysS carrying the hGMPK plasmid was used to inoculate 1 l of lactose-containing auto-inducing medium. hGMPK was obtained as a catalytically active monomer upon applying different purification steps. The monomer peak of hGMPK was pooled, concentrated to 38 mg/ml, aliquoted, and then stored at –80 °C. The Bradford dye-binding assay was used to determine the hGMPK concentration.

### Enzyme activity assay

The enzymatic activity of hGMPK was determined by a standard NADH-dependent lactate dehydrogenase/pyruvate kinase-coupled assay using a JASCO V-650 UV–Vis spectrophotometer (Konrad 1992). All measurements were performed at 25 °C in the assay buffer, which consisted of 100 mM Tris-HCl, pH 7.5, 100 mM KCl, and 10 mM MgCl_2_. The final hGMPK concentration in the assay was 18 nM.

### Home source SAXS data acquisition

SAXS data was collected at the home-built SAXS apparatus (Quevedo et al. 2008) to check the effect of various hGMPK concentrations. The hGMPK samples and corresponding buffers in different experimental conditions were exposed and measured under stationary conditions in the assembled quartz capillary flow cell with 1.0 mm diameter. Potential radiation damage in the measured samples was checked with SDS-PAGE and the kinase activity assay. In this way, we verified that the enzyme did not suffer from radiation damage during X-ray exposures.

### Synchrotron SAXS data acquisition

hGMPK samples and their matching buffers under different experimental conditions were filled in separate 1.0-mm-diameter borosilicate capillaries and sealed with capillary wax for SAXS measurements. SAXS data was collected at the coherent SAXS beamline X12SA, Swiss Light Source (SLS) at the Paul Scherrer Institute (PSI, Switzerland). The photon flux was ~10^12^ photons*/*s at the sample position. The photon energy was 12.4 keV, which corresponds to a wavelength of 1.0 Å. X-ray images were acquired with an integration time of 100 ms per image.

The X-ray spot size on the sample was 100 × 100 µm and the X-ray beam was focused on the detector. SAXS images were acquired using a PILATUS 2M detector at a sample-detector distance of ~7 m. Images were acquired at three different spots on the capillary; these observation spots were 1.0 mm apart and were centered across the capillary.

### SAXS data processing and analysis

Scattering curves were obtained from the raw PILATUS detector images using MATLAB (R2012a) and further processed with Origin (8.6) and ATSAS (2.4) software (Konarev et al. 2006; Petoukhov et al. 2012). hGMPK scattering curves *I*(*q*) vs*. q* were plotted after the subtraction of their respective buffer from the hGMPK samples as a function of momentum transfer *q* (Å^−1^), which is given by:

1 $$ q = \frac{4\pi \sin \theta }{\lambda }, $$

where 2*θ* is the scattering angle and *λ* is the X-ray wavelength in Å (Glatter and Kratky 1982; Guinier and Fournet 1955).

The SAXS scattering curves were then analyzed with different parameters. The Guinier approximation for a monodisperse sample of globular protein can be used to describe the X-ray scattering at low *q* as:

2 $$ \ln \left[ {I\left( q \right)} \right] = \ln \left[ {I\left( 0 \right)} \right] - \left( {\frac{{R_{\text{g}}^{2} }}{3}} \right) \times (q^{2} ), $$

where *R*_g_ is the radius of gyration and *I*(*0*) is the forward scattered intensity (Glatter and Kratky 1982; Guinier and Fournet 1955). The Guinier approximation is valid for the *qR*_g_ ≤ 1.3 region. *R*_g_ is essentially the second moment of the distribution of shape and size of proteins about the mean and its calculation requires electron density rather than mass as a weighing factor. For this *q* range, the Guinier plot will give a straight line from which *R*_g_ and *I*(*0*) values are extracted. *R*_g_ values were determined by various procedures including manual Guinier plot (not shown), PRIMUS and GNOM (Konarev et al. 2003; Svergun et al. 1995). The *I*(*0*) value is directly proportional to the multiplication of molar protein concentrations and molecular mass of the scattering hGMPK molecule. *I*(*0*) was approximated by the extrapolation of SAXS intensity to *q* = 0. The Kratky plots (*I*(*q*) × *q*^*2*^ vs*. q*) were then used to assess the globular or Gaussian chain-like nature of hGMPK under different experimental conditions.

ATSAS suite was used to reconstruct and analyze the three-dimensional SAXS models. The detailed procedure is presented in the supplementary information. The inertial axes of the SAXS data-based averaged hGMPK models were then overlaid to different crystal structures or to the different SAXS models by the SUPCOMB13 software (Kozin and Svergun 2001). The open-source software PyMOL was used for graphical analysis and for figure preparation.

### CD measurements of different hGMPK conformations

CD measurements were performed to confirm the observed size and shape changes in different hGMPK conformations with SAXS. hGMPK concentration was 80 µM. The buffer was 10 mM potassium phosphate pH = 7.5, 2 mM MgSO_4_, and 0.5 mM Tris (2-carboxyethyl) phosphine (TCEP). Substrate (GMP + AMP-PNP and GMP + ADP) concentration was 1 mM in hGMPK samples and buffer. The bi-substrate analog Ap5G was used at 100 µM concentration.

CD spectra were measured separately for hGMPK samples and respective buffers at 25 °C between 180 and 300 nm. Five scans were collected at 1 s/measurement and then averaged. The averaged CD spectra for an hGMPK conformation and its respective buffer was subtracted and then smoothed (window size = 3). The CD spectra were finally plotted with the help of Origin 8.6.

## Results and discussion

**Fig. 2:**
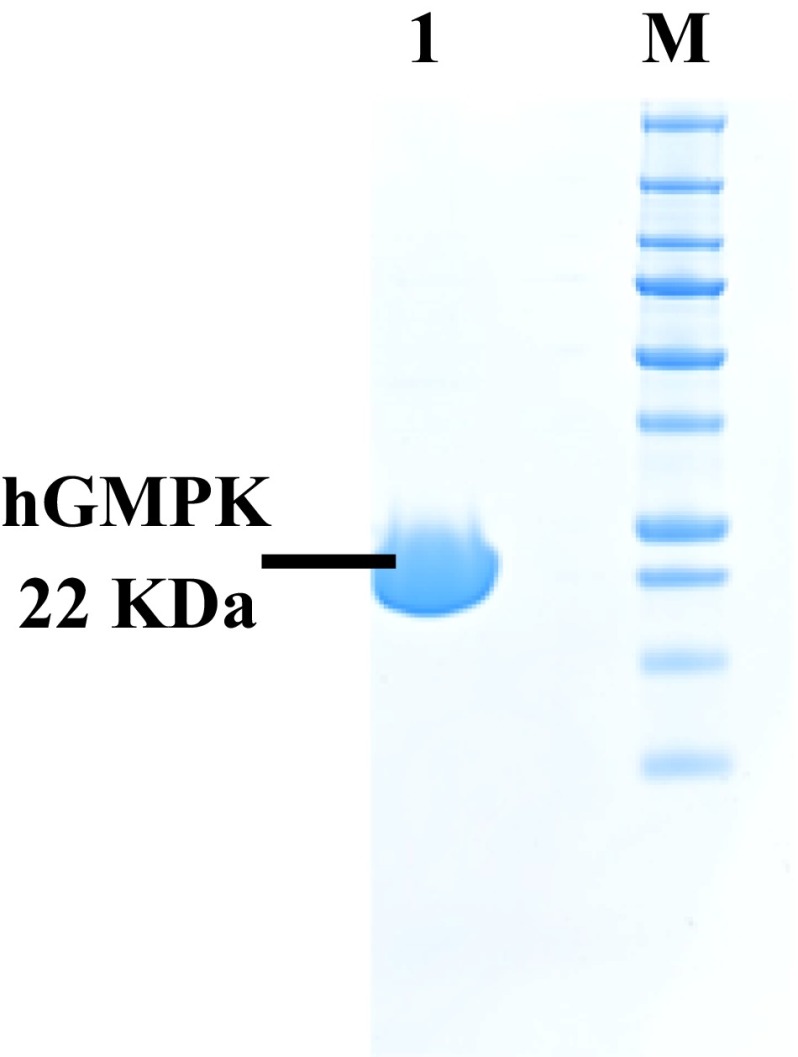
Purified recombinant hGMPK is catalytically active. Bacterially produced hGMPK was purified by affinity and gel filtration chromatography in two successive steps. Purified hGMPK is a single band on 12 % SDS-PAGE

Human guanylate kinase, hGMPK, was expressed in *E. coli*, and the histidine-tagged protein (~22 kDa) was purified via nickel agarose-affinity chromatography and size-exclusion chromatography in successive steps (Fig. 2a). The purified recombinant hGMPK protein was catalytically active as determined in the NADH-dependent enzyme-coupled assay and yielded the kinetic parameters shown in Table 1. The activity of recombinant hGMPK presented in this work is in contrast to previous reports where hGMPK was found inactive (Ardiani et al. 2009; Brady et al. 1996).

**Table 1:** Kinetic parameters obtained from the steady-state kinetic assay measuring hGMPK activity (*T* = 25 °C). The ATP:GMP phosphoryl transferase activity of purified recombinant hGMPK was determined by using the NADH-dependent enzyme-coupled assay

Substrate	*k*_cat_ (s^−1^)	*k*_*m*_ (µM)	*k*_cat_ */K*_*m*_ (M^−1^ s^−1^)
GMP	79	25	3.2 × 10^6^
Mg-ATP	79	95	0.8 × 10^6^

**Fig. 3:**
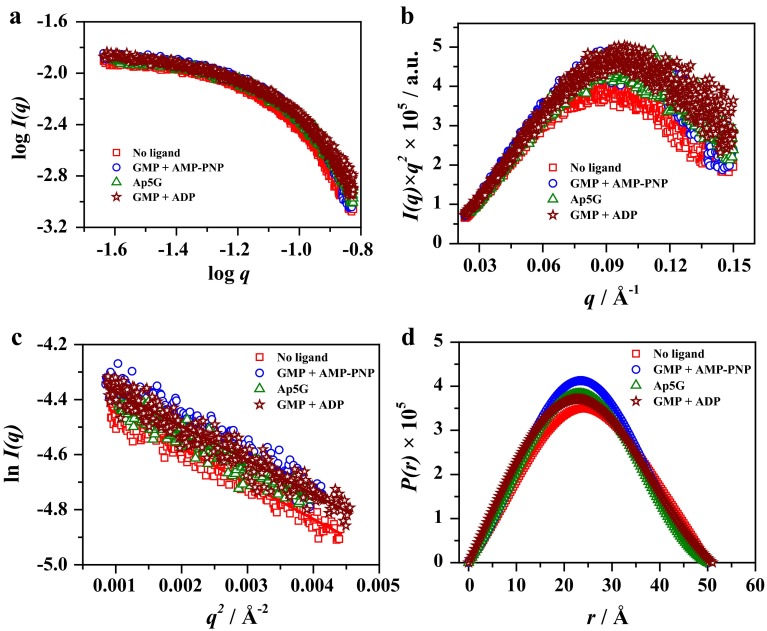
SAXS data analysis for open and completely closed hGMPK conformations. **a** SAXS intensity curves. **b** Kratky profiles. **c** Guinier plots. **d** Computed pair distance distributions

The radius of gyration (*R*_g_) and forward scattered scattering (*I*(*0*)) of hGMPK remained unchanged for increasing concentrations (6.5–26.5 mg/ml) in the home SAXS measurements (data not shown). Also, the hGMPK activity did not decrease after X-ray exposure during SAXS measurements (data not shown). In the following step, different hGMPK conformations in the presence and absence of substrates and analogs were measured at the third-generation cSAXS beamline: No ligand (open form), GMP + AMP-PNP (completely closed form 1), Ap5G (completely closed form 2), and GMP + ADP (completely closed form 3). The SAXS measurements were performed for corresponding buffer solutions as well. SAXS measurements of different hGMPK conformations along with buffer were free from radiation damage. This was checked by plotting integral X-ray intensity of consecutive scattering images at an observation spot with respect to the X-ray exposure time (Fig. S2, ESM). The buffer-subtracted SAXS intensity profiles of different hGMPK conformations were plotted and analyzed with various SAXS parameters to evaluate structural changes in different hGMPK conformations (Fig. 3a). The bell-shaped Kratky profiles depict a globular nature for all measured hGMPK conformations (Fig. 3b).

The unliganded form of hGMPK was found to be monomeric by comparing its elution profile with standard proteins by size-exclusion chromatography (Fig. S1 and Table S1, ESM). Open and completely closed hGMPK conformations (1–3) have identical *I*(*0*) values (Table 2). This implies that hGMPK is a monomer in all investigated conformations and the binding of substrates and analogs did not cause its oligomerization. Different slopes of manual Guinier plots for hGMPK conformations indicate variation in the hGMPK size (Fig. 3c). The size (*R*_g_) of different hGMPK conformations was calculated by pair distance distribution curves (Table 2). The open hGMPK conformation is bigger in size (*R*_g_ ~ 20.1 (± 0.04) Å) than the completely closed hGMPK conformations: GMP + AMPPNP (1, *R*_g_ ~ 18.4 (± 0.04) Å), Ap5G (2, *R*_g_ ~ 18.1 (± 0.03) Å) and GMP + ADP (3, *R*_g_ ~ 18.4 (± 0.04) Å). In tendency, the completely closed hGMPK conformations (1–3) get compact in comparison to the open hGMPK conformation. The maximum dimensions *D*_max_ for different hGMPK conformations were also calculated with the pair distance distribution curves (Fig. 3d; Table 2). The maximum dimensions of open and completely closed hGMPK conformations (1–3) are similar (*D*_max_ ~ 50 Å) (Table 2).

**Table 2:** Structural parameters for different hGMPK conformations as derived with SAXS measurements

hGMPK sample	Conformation	*I*(*0*) × 10^−2^	*R*_g_ (Å)	*D*_max_ (Å)
Apo-form	Open	1.3	20.1 (± 0.04)	50.0
GMP + AMP-PNP	Completely closed 1	1.4	18.4 (± 0.04)	50.0
Ap5G	Completely closed 2	1.4	18.1 (± 0.03)	50.0
GMP + ADP	Completely closed 3	1.4	18.4 (± 0.04)	51.0

The overall fold of hGMPK is very similar to that of other members of the NMP-kinase family, in particular to mGMPK and yGMPK (Blaszczyk et al. 2001; Kandeel and Kitade 2011; Sekulic et al. 2002). The 197-amino-acid-long hGMPK is just one amino acid shorter at the C-terminus than the closely related mGMPK, with which it has high amino acid sequence identity (88 %). The crystal structures of open yGMPK [apo, *pdb 1EX6* (Blaszczyk et al. 2001)] and partially closed yGMPK [GMP, *pdb 1EX7* (Blaszczyk et al. 2001)] are also available. All available GMPK structures share three major structural domains: the NMP-BD, a CORE domain, and a LID domain, which are interconnected by four dynamic hinges. In its apo-form (yGMPK without ligand), the NMP-BD and the LID domain are farthest apart, and therefore the open form of yGMPK is more extended than the partially closed form of yGMPK (Blaszczyk et al. 2001). The binding of GMP to the NMP-BD in yGMPK (in complex with GMP) causes a significant movement of the NMP-BD towards the LID domain with comparatively smaller movement of the LID domain in the same direction. Therefore, GMP binding brings the NMP-BD and the LID domain closer to each other resulting in a compaction of the partially closed yGMPK (GMP-complexed) as compared to its open yGMPK (apo) form (Blaszczyk et al. 2001). The simultaneous binding of both substrates (GMP + ADP) in mGMPK brings the NMP-BD and LID domain closer together than in the partially closed yGMPK (GMP) conformation (Sekulic et al. 2002). Therefore, the fully closed conformation of GMPK is the most compact conformation (Sekulic et al. 2002). Also, the open form of yGMPK is the most flexible conformation, while the fully closed hGMPK form is the most rigid conformation (Blaszczyk et al. 2001; Sekulic et al. 2002).

**Fig. 4:**
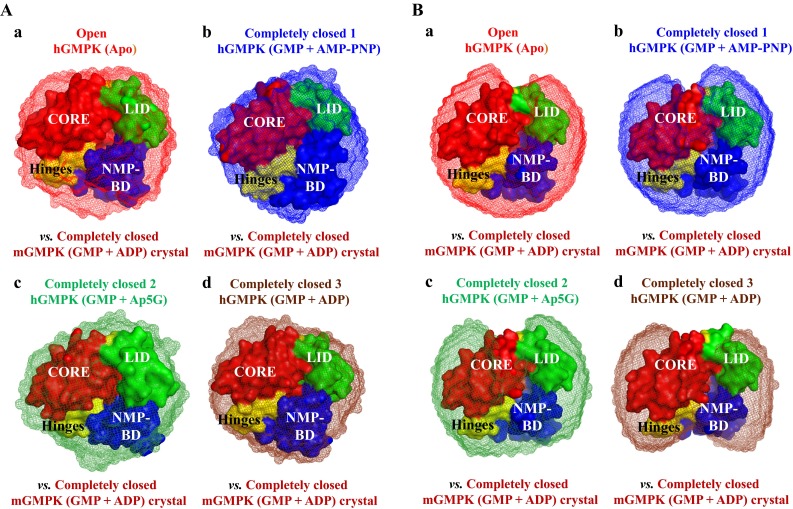
Overlay of reconstructed hGMPK SAXS models (mesh representation) on the crystal structure of the completely closed mGMPK conformation (GMP + ADP, *pdb 1LVG* (Sekulic et al. 2002), surface representation). GASBOR (**A**) and DAMMIN (**B**) SAXS models of different hGMPK conformations are compared separately. **a** Open hGMPK conformation (apo-form). **b** Completely closed hGMPK conformation 1 (GMP + AMP-PNP). **c** Completely closed hGMPK conformation 2 (Ap5G). **d** Completely closed hGMPK conformation 3 (GMP + ADP). Overlay was done with SUPCOMB13 and the resulting NSD values are shown in Table S4, ESM. Different structural regions in the crystal structure of completely closed mGMPK (NMP-BD, CORE, and LID) and hinges are color-coded

Changes in the global structure of hGMPK from open to completely closed conformation were investigated by reconstructing low resolution SAXS models within the shape constraints computed with *P*(*r*) function analysis. Structural interpretation for each hGMPK conformation was performed with 10-15 reconstructed SAXS models (*pdb* files) having low *χ*^2^ values between experimental scattering intensity and computed scattering intensity for the GASBOR models (*χ*^2^ = 1.6–2.6) or DAMMIN models (*χ*^2^ = 1.5–2.2) (Table S2, ESM). The SAXS models generated with GASBOR or DAMMIN programme were averaged separately by DAMAVER (Fig. S2, ESM). The alignment and averaging procedure for the reconstructed SAXS models was scrutinized with normalized spatial discrepancy (NSD) values. DAMMIN and GASBOR models with higher NSD values (NSD > mean + 2 × variation) were discarded (Table S2, ESM). To identify the structural changes in the hGMPK molecule, the averaged SAXS models of different hGMPK conformations were overlaid on the crystal structure of the fully closed mGMPK form [*pdb 1LVG* (Sekulic et al. 2002)] (Fig. 4, Tables S3, S4, ESM). With respect to the NSD values, the overlays were sufficient and revealed compactness from open to completely closed hGMPK conformations (Fig. 4, Tables S3, S4, ESM).

The overall fold of hGMPK is very similar to that of other members of the NMP-kinase family, in particular to mGMPK and yGMPK (Blaszczyk et al. 2001; Kandeel and Kitade 2011; Sekulic et al. 2002). The 197-amino-acid-long hGMPK is just one amino acid shorter at the C-terminus than the closely related mGMPK, with which it has high amino acid sequence identity (88 %). Therefore, the in silico homology model of the completely closed form of hGMPK was constructed by using the fully closed form of mGMPK (Sekulic et al. 2002) as a template (Fig. 1). In addition to the completely closed mGMPK crystal structure (*pdb 1LVG*), the substrate-induced conformational changes in hGMPK SAXS models were compared with the homology model of the completely closed form of hGMPK (Tables S3, S4, ESM). The overlay of different hGMPK SAXS models on the completely closed hGMPK homology model is similar to that of the completely closed mGMPK crystal structure (not shown). 

Three-dimensional reconstructions are globular for the open and completely closed hGMPK conformations (Fig. 4). There are small but distinguishable differences in shape between open and completely closed hGMPK conformations. The open hGMPK conformation is characteristic of the unliganded enzyme. The binding of GMP (substrate) to the NMP-BD and AMP-PNP (ATP analog) to the LID domain induces the completely closed hGMPK conformation 1. The binding of the bi-substrate analog Ap5G to NMP-BD and LID domain induces the completely closed hGMPK conformation 2. The binding of GMP (substrate) to the NMP-BD and ADP (reaction product) to the LID domain induces the completely closed hGMPK conformation 3. The completely closed hGMPK conformations are smaller in size than those of the open hGMPK conformation (Fig. 4).

**Fig. 5:**
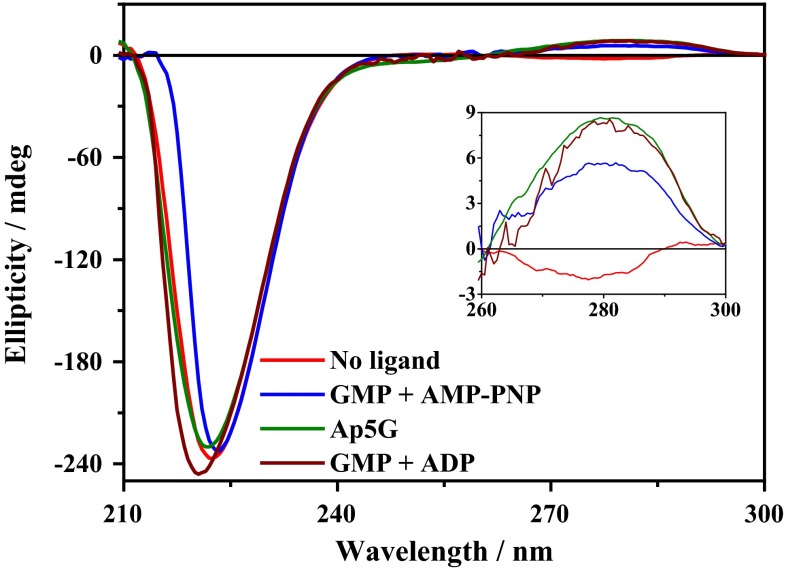
CD spectrums of different hGMPK conformations

The compaction observed from open to completely closed hGMPK conformations was compared with small differences in the size between crystal structures of open yeast conformation (*pdb 1LVG*), partially closed conformation (*pdb 1EX6*) and completely closed mGMPK conformation (*pdb 1EX7*). The open to completely closed hGMPK conformation follows the trend observed in the size reduction from open yeast conformation (*R*_g_ ~ 18.8 Å) to the partially closed yeast GMPK conformation (*R*_g_ ~ 17.8 Å) and to the completely closed mGMPK conformation (*R*_g_ ~ 17.2 Å) (Tables S5, ESM). One way to explain the compaction between open hGMPK and completely closed hGMPK could be the closeness of the NMP-BD and ATP-binding domains in comparison to the open hGMPK conformation. The observed structural changes in the size and shape of open and closed hGMPK conformations were confirmed by circular dichroism (CD) spectroscopy. CD spectroscopy is a valuable technique for the investigation of structural changes in proteins caused by the binding of ligands (Fasshauer et al. 2002; Greenfield 2006; Petri et al. 2012). The differences in secondary structures from open to completely closed hGMPK conformations (1–3) observed with CD spectra are small and present at 210–240 nm (peptide bonds) and 260–300 nm (tertiary structure) (Fig. 5). Hence, the compaction in hGMPK structure during catalytic reaction (open to completely closed) could result from a change in the secondary structure content of hGMPK.

In summary, our SAXS analyses have tracked the missing structural differences in the size and shape of open and completely closed hGMPK conformations. The open-to-close conformational transition of hGMPK brought about by the sequential binding of its substrates supports the induced fit enzymatic mechanism. The good fitting of the completely closed mGMPK crystal structure and completely closed hGMPK SAXS models indicates the conservation of the 3D-fold of the guanylate kinase domain in these two enzymes.

## Electronic supplementary material

Supplementary material 1 (DOCX 1998 kb)

## Abbreviations

ADP: Adenosine 5′- diphosphate
AKs, AMP kinases: Adenylate kinases
AMP-PNP: Adenosine 5′-(β, γ-imido) triphosphate, non-hydrolysable ATP analog
Ap5G: P^1^ -(5' -Adenosyl) P^5^ -(5' -guanosyl) pentaphosphate, non-hydrolysable bi-substrate analog
cGMP: 3’, 5’ -cyclic guanosine monophosphate
DTT: Dithiothreitol
GMP: Guanosine 5’ -monophosphate
GDP: Guanosine 5’ -diphosphate
GTP: Guanosine 5’ -triphosphate
HEPES: 4-(2-hydroxyethyl)piperazine-1-ethanesulfonic acid
hGMPK: Human guanylate kinase
hGMPK (apo): Open hGMPK form (ligand-free)
hGMPK (GMP + AMP-PNP): Completely closed hGMPK form 1
hGMPK (Ap5G): Completely closed hGMPK form 2
hGMPK (GMP + ADP): Completely closed hGMPK form 3
mGMPK: Mouse guanylate kinase
NMP kinases, NMPKs: Nucleoside monophosphate kinases
NMP-BD: NMP binding domain
PSI: Paul Scherrer Institute
SAXS: Small-angle X-ray scattering
SDS-PAGE: Sodium dodecyl sulfate–Polyacrylamide gel electrophoresis
SLS: Swiss Light Source
SUMO: Small ubiquitin-related modifier
yGMPK: Yeast guanylate kinase

## List of symbols

*D*_max_: Maximum dimensions
*I*(*q*): X-ray scattering intensity
*I*(*0*): Forward scattered intensity
*P*(*r*): Pair distance distribution function
*Pdb*: Protein Data Bank file format
pH/pH scale: Hydronium ion concentration measurement
*q*: Momentum transfer
*R*_g_: Radius of gyration
