# Supplementary material for: Insights into open/closed conformations of the catalytically active human guanylate kinase as investigated by small-angle X-ray scattering
Source: Eur Biophys J. 2015 Oct 7;45:81–9. doi: 10.1007/s00249-015-1079-9 (PMC4698301; doi:10.1007/s00249-015-1079-9)
Supplement: Supplementary file 1 — Supplementary material 1 (DOCX 1998 kb) [file 249_2015_1079_MOESM1_ESM.docx]

**Supplementary Information**

**Insights into open/closed conformations of the catalytically active human guanylate kinase as investigated by small-angle X-ray scattering**

**Rohit Jain^1, *^, Nazimuddin Khan^1 *^, Andreas Menzel^2^, Ivan Rajkovic^2^, Manfred Konrad^1, ##^ and Simone Techert^1, #^**

1 = Max Planck Institute for Biophysical Chemistry,

Am Fassberg 11, 37077 Göttingen, Germany.

2 = Paul Scherrer Institute, 5232 Villingen, Switzerland.

* = contributed equally.

^#^ = corresponding author.

^#^ E-mail: stecher@mpibpc.mpg.de; simone.techert@desy.de

^##^ E-mail: mkonrad@mpibpc.mpg.de

Rohit Jain: Max Planck Institute of Biophysical Chemistry, Am Fassberg 11, 37077 Göttingen, Germany.

Dr. Manfred Konrad: Max Planck Institute of Biophysical Chemistry, Am Fassberg 11, 37077 Göttingen, Germany.

Nazimuddin Khan: Max Planck Institute of Biophysical Chemistry, Am Fassberg 11, 37077 Göttingen, Germany.

Dr. Andreas Menzel: Paul Scherrer Institute, 5232 Villingen, Switzerland.

Dr. Ivan Rajkovic: Paul Scherrer Institute, 5232 Villingen, Switzerland.

Prof. Dr. Simone Techert: Max Planck Institute of Biophysical Chemistry, Am Fassberg 11, 37077 Göttingen, Germany.

Institute for X-ray Physics at University of Göttingen, Friedrich-Hund-Platz 1, 37077 Göttingen, Germany.

FS-SCS at DESY, Notkestraße 85, 22607 Hamburg, Germany.

**Supplementary materials and methods**

Cloning of human guanylate kinase

The 591 bp open reading frame (ORF) of hGMPK (UniProt entry Q16774, also termed GMP kinase, GUK1, or GMK), was amplified via polymerase chain reaction (PCR) using the previously described DNA template ([Kuhlendahl et al. 1998](#_ENREF_5); [Kumar et al. 2000](#_ENREF_6); [Prinz et al. 1999](#_ENREF_8)). NdeI and BamHI sites (*underlined*) were incorporated into the two gene-specific forward (F) and reverse (R) primers used (IBA GmbH, Göttingen):

hGMPK-F, 5´-GGGAATTCCATATGTCGGGCCCCAGGCCTGTGG-3´ and

hGMPK-R, 5´-CGCGGATCCTTAGGCGCCGGTCCTTTGAGCTTTCTTG-3´.

The PCR product was gel-purified, digested with NdeI and BamHI-HF (New England Biolabs), and ligated overnight at 16 °C into the pET14b-SUMO∆Thr vector ([Hazra et al. 2010](#_ENREF_4)) using T4 DNA ligase (New England Biolabs). The ligation mixture was used to transform *E. coli* XL1-Blue cells. Positive clones were identified following restriction digestion of isolated plasmid DNA with NdeI and BamHI-HF, and ultimately the entire gene insert was sequenced. The final construct includes an N-terminal hexa-histidine (His_6_) tag followed by the SUMO (small ubiquitin-related modifier; SUMO family protein SMT3 of 101 residues)-tag, which was used to improve heterologous protein solubility and stability ([Panavas et al. 2009](#_ENREF_7)). For overproduction of the enzyme, the *E. coli* expression strain BL21-(DE3)-pLysS was used.

Expression and purification of human guanylate kinase

One liter of lactose-containing auto-inducing media was inoculated with the hGMPK plasmid carrying BL21-(DE3)-pLysS starter culture. This culture was incubated at 37 °C with rapid shaking until the optical density at 600 nm reached about 0.7 and was then transferred to 21 °C for overnight induction. *E. coli* cells were harvested, centrifuged, and the pellet was re-suspended in lysis buffer (50 mM HEPES, pH 8.0, 300 mM NaCl, 0.5% Triton X-100, 5 mM DTT, containing a cocktail of protease-inhibitors) and lysed by sonication. The lysate was cleared by centrifugation at 10,000g for 1 h at 4 °C. The supernatant was collected and subjected to batch purification by nickel-resin affinity chromatography.

One gram of Protino Ni-IDA resin (Macherey-Nagel) was added to this supernatant in a centrifugation tube, and the suspension was agitated on an orbital shaker for 1 h at 4 °C. The resin was washed four times with 40 ml of buffer A (50 mM HEPES, pH 8.0, 300 mM NaCl, 15 mM imidazole and 2 mM DTT) each time with 25 min incubation on an orbital shaker at 4 °C. The resin was transferred to an empty chromatography column (5 ml column bed, Thermo Scientific), and the His_6_-SUMO fusion hGMPK was eluted with buffer B (50 mM HEPES, pH 8.0, 150 mM NaCl, 0.1% Triton X-100, 250 mM imidazole and 2 mM DTT). This yielded 110 mg of His_6_-SUMO-hGMPK fusion protein as determined by the Bradford dye-binding assay. The His_6_-SUMO tag was then cleaved by SUMO-protease (1:100 M ratios) at room temperature for 30 min. To remove imidazole from the sample, the solution was passed through a PD10 column (Sephadex-G25) using buffer C (25 mM HEPES, pH 7.5, 300 mM NaCl, and 2 mM DTT). 500 mg of Protino Ni-IDA resin was added to remove the His_6_-SUMO tag, and the resin suspension was incubated for 30 min on an orbital shaker at 4 °C. The resin suspension was passed through an empty column (5 ml column bed, Thermo Scientific) with a filter frit to collect hGMPK without His_6_-SUMO tag in the flow-through.

To further purify the homogeneous enzyme, eluted hGMPK was loaded on a Buffer C pre-equilibrated Superdex-75 10/300 GL gel filtration column (GE Healthcare). hGMPK eluted as a monomer as indicated by the elution profile of marker proteins (BioRad Gel Filtration Standard). The monomer peak of hGMPK was pooled, concentrated to 38 mg/ml, aliquoted, and then stored at -80 °C. The Bradford dye-binding assay was used to determine hGMPK concentration.

hGMPK was found to be monomeric by comparing its elution profile with standard proteins (Supplementary Fig. S1 and Supplementary Table S2). The Superdex 75 10/300 GL column was first equilibrated in 50 mM potassium phosphate and 150 mM NaCl (pH 6.8, 0.3 ml/min flow rate, at 25 ^o^C). Standard proteins were run and their elution profile was recorded (Supplementary Fig. S1b, c and Supplementary Table S2). The calibration curve for Superdex 75 10/300 GL column was determined as:

$$K_{d}={(V_{E}- V_{0})}/{\left( V_{T}- V_{0} \right), 1}$$

where $K_{d}$ is the distribution coefficient, $V_{E}$ is the elution volume of protein, $V_{0}$ is the void volume of column ($V_{0}=8 ml$), and $V_{T}$ is the total volume of the packed column bed ($V_{T}=24 ml$). Absorbance was monitored at 280 nm using ÄKTA prime plus (GE Healthcare Life Sciences). $V_{E}$ for hGMPK was 13.9 ml. The relative molecular mass of hGMPK was found to be 22 kDa which represents its monomeric form.

SAXS data analysis:

CRYSOL programme available from the ATSAS suite was used to calculate the theoretical solution scattering amplitudes from known GMPK crystal structures. They were fitted to the measured X-ray scattering profile of hGMPK under different experimental conditions ([Svergun et al. 1995](#_ENREF_11)). CRYSOL provided the $\chi^{2}$ values for the estimation of discrepancy between theoretical and experimental curves ([Svergun et al. 1995](#_ENREF_11)).

CRYSOL ([Svergun et al. 1995](#_ENREF_11)) first calculates theoretical scattering intensity, $I \left( q, r_{0},\delta\rho\right)$ for the solvent density value ($\rho_{o}=334 e {nm}^{-3}$) which corresponds to the bulk water with the default parameters ($r_{0}= r_{m} and \delta\rho=30 {nm}^{-3}$). Here, $r_{m}= N^{-1} \sum_{j=1}^{N} r_{gj}$ is the actual average radius of the atomic group, and$r_{0}$, the effective atomic radius, is a variable parameter that can be used to change the displaced volume per atomic group and thus to adjust the total excluded volume. $\delta\rho$, is the unitary density. For proteins, $r_{m}$ is normally around 0.162 nm.

CRYSOL ([Svergun et al. 2001](#_ENREF_12)) adjusts parameters to fit the experimental scattering intensity $I_{exp}\left( q \right)$. It makes a plain grid search for $0.96 r_{m} \leq r_{0} \leq1.04 r_{m} and 0 \leq\delta\rho\leq6e {nm}^{-3}$ to minimize the function:

$$\chi^{2}\left( r_{0}, \delta\rho\right)=\frac{1}{N_{p}} \sum_{i=1}^{N_{p}} \left[ \frac{I_{exp}\left( q_{i} \right)-cI(q_{i}, r_{0},\delta\rho)}{\sigma(q_{i})} \right]^{2}, 2$$

where $N_{p}$ is the number of experimental points, the $\sigma(q_{i})$ are the experimental errors and

$$c= \left[ \sum_{i=1}^{N_{p}} \frac{I_{exp}\left( q_{i} \right)I(q_{i}, r_{0},\delta\rho)}{{\sigma(q_{i})}^{2}} \right]\left[ \sum_{i=1}^{N_{p}} \frac{I{(q_{i}, r_{0},\delta\rho)}^{2}}{{\sigma(q_{i})}^{2}} \right]^{-1} 3$$

is the scaling factor. The fit is presented on a graphic display and the parameters can be changed manually by the user.

Another important function for SAXS measurements is the pair distance distribution function *P(r)* which is the radially averaged autocorrelation function for the derivation of the electron pair distance distribution function of the interatomic vectors within the hGMPK molecule. It is plotted with respect to the real space radius *r*  (Å) as:

$$P\left( r \right)=\frac{1}{2\pi} \int I\left( q \right) q \times r\sin\left( q \times r \right)dq , 4$$

The *P(r)* function is a histogram of the frequency of vector lengths where small volume elements are connected within the entire volume of the scattering particle. It is required for *ab initio* reconstructions and estimated to be zero at *r* = 0 and *r* = *D_max_* , where *D_max_* is the maximum linear dimension of the scattering molecule ([Glatter and Kratky 1982](#_ENREF_2); [Guinier and Fournet 1955](#_ENREF_3)). . The *P(r)* function was obtained through the indirect Fourier transformation of the experimental SAXS scattering curve by the ATSAS programme GNOM which uses a regularization multiplier to balance the smoothness of the trial *P(r)* functions with the goodness of fit to the experimental data ([Svergun 1992](#_ENREF_10)). The three-dimensional molecular SAXS surfaces were then reconstructed for the detailed description of hGMPK under different experimental conditions by *ab initio* modeling with DAMMIN and GASBOR ([Svergun et al. 2001](#_ENREF_12)). 10-15 independent DAMMIN envelopes were generated without any predefined shape or symmetry bias and then averaged by DAMAVER to avoid problems due to the over-fitting of the experimental data and to ensure the reproducibility of modelling ([Volkov and Svergun 2003](#_ENREF_13)). GASBOR envelopes were also averaged with DAMAVER.

**Supplementary Figures:**

**a.**

**b.**

**c.**


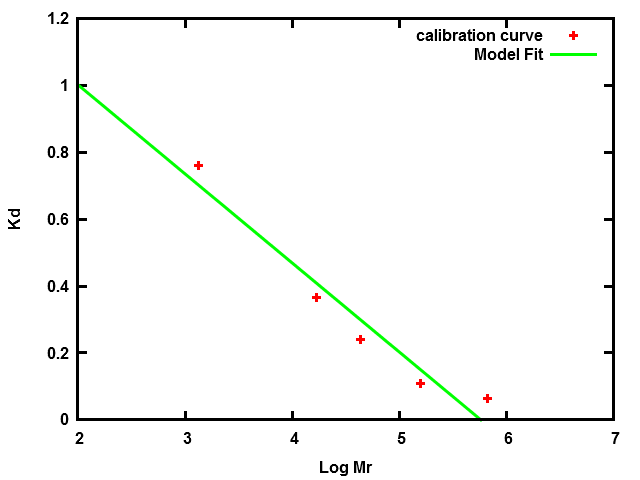


**hGMPK monomer**

**(~ 22 kDa)**

Figure S1. Recombinant hGMPK is monomer and its relative molecular mass is 22 KDa in solution. (a) hGMPK elutes as a monomer peak. (b) Elution of standard proteins on Superdex 75 10/300 GL column. (c) The relative molecular mass of hGMPK was determined to be 22 KDa with the calibration curve for standard proteins eluted on Superdex 75 10/300 GL column. Please see supplementary materials and methods for details.

Figure S2. The X-ray scattering data used for the analysis of different hGMPK conformations is free from radiation damage. The unliganded hGMPK conformation is shown here as an example. The integrated X-ray intensity (q = 0.15 × 10^-3^ – 0.18 Å^-1^) of consecutive scattering images at different observation spots on the quartz capillary is plotted with respect to the X-ray exposure time.


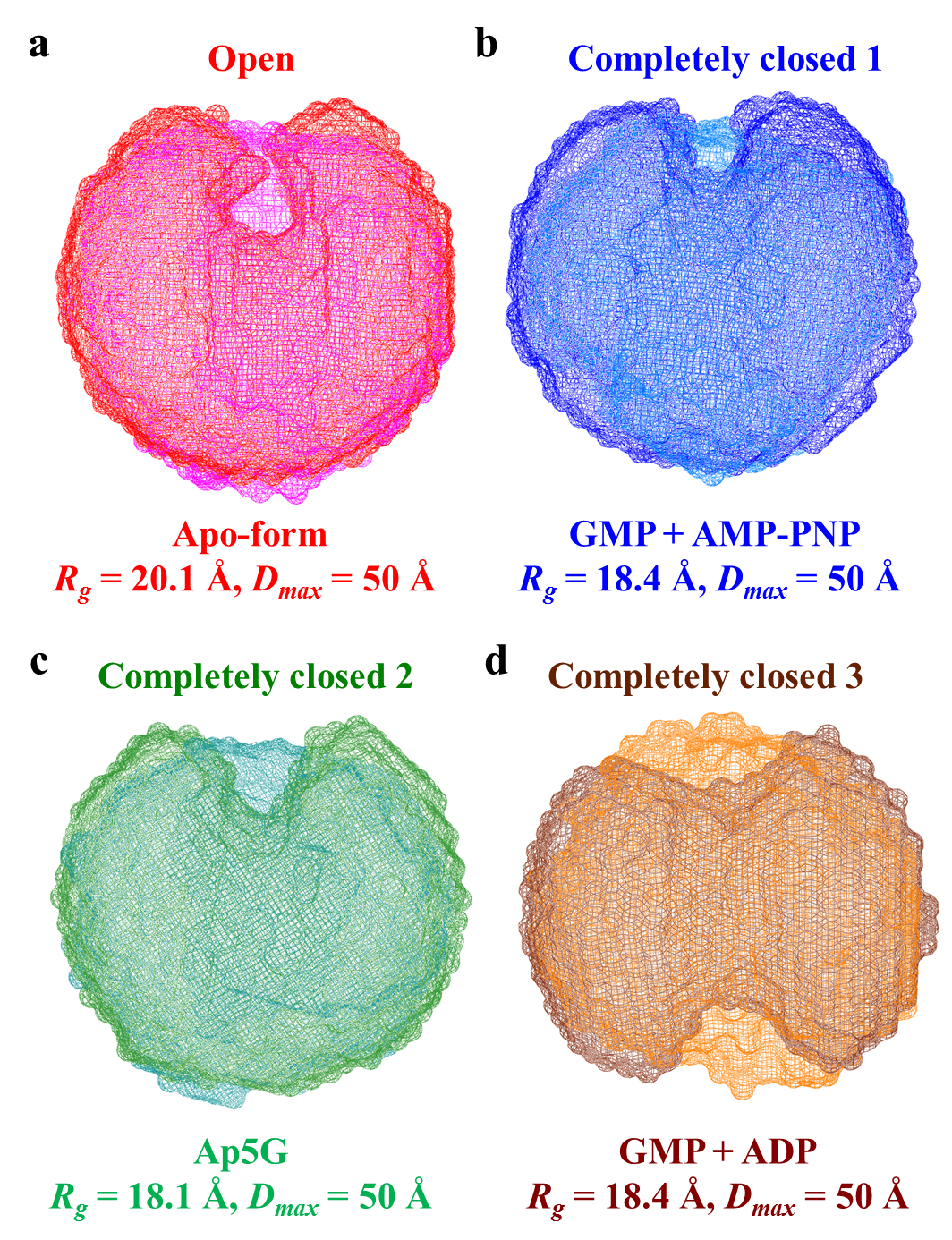


Figure S3. DAMMIN and GASBOR SAXS data-based averaged models of different conformations of hGMPK bound to different substrates and analogs showing their size (*R_g_*) and maximum dimensions (*D_max_*) values. (a) Open conformation (apo-form). (b) Completely closed conformation 1 (GMP + AMP-PNP). (c) Completely closed conformation 2 (Ap5G). (d) Completely closed conformation 3 (GMP + ADP).

**Supplementary tables:**

| **Protein** | **Molecular weight**  **(KDa)** | **Integrated peak area**  **(mU × min)** | **Retention time**  **(min)** |
| --- | --- | --- | --- |
| Bovine Thyroglobulin | 670 | 193.4 | 30.0 |
| Bovine γ-globulin | 158 | 297.4 | 32.3 |
| Chicken Ovalbumin | 44 | 698.5 | 39.3 |
| **Human**  **Guanylate Kinase** | **22** | **243.9** | **46.3** |
| Horse Myoglobin | 17 | 439.9 | 46.3 |
| Vitamin B_12_ | 1.35 | 690.7 | 67.14 |

Table S1: The elution profile of hGMPK and standard proteins on Superdex 75 10/300 GL column.

| **hGMPK**  **sample** | **Conformation** | **GASBOR** | | **DAMMIN** | |
| --- | --- | --- | --- | --- | --- |
|  |  | $\chi^{2}$ | **NSD** | $\chi^{2}$ | **NSD** |
| **apo-form** | **open** | **1.6** | **1.0 ±**  **0.01** | **1.5** | **0.5 ±**  **0.05** |
| **GMP +**  **AMP-PNP** | **completely closed 1** | **1.9** | **0.9 ±**  **0.01** | **1.8** | **0.6 ±**  **0.09** |
| **Ap5G** | **completely closed 2** | **1.9** | **0.9 ±**  **0.01** | **1.8** | **0.5 ±**  **0.02** |
| **GMP +**  **ADP** | **completely closed 3** | **2.6** | **1.0 ±**  **0.01** | **2.2** | **0.5 ±**  **0.01** |

Table S2. Modeling parameters of SAXS models for different hGMPK conformations. NSD = Normalized spatial discrepancy.

| **hGMPK**  **SAXS model**  **(GASBOR)** | **Conformation** | **NSD value mGMPK**  **(GMP + ADP)**  **(*pdb 1LVG)*** | **NSD value hGMPK**  **(homology**  **model)** |
| --- | --- | --- | --- |
| **apo-form** | **open** | **1.0** | **1.0** |
| **GMP +**  **AMP-PNP** | **completely closed 1** | **1.0** | **1.0** |
| **Ap5G** | **completely closed 2** | **0.9** | **0.9** |
| **GMP + ADP** | **completely closed 3** | **1.0** | **1.0** |

Table S3. Normalized spatial discrepancy (NSD) values for the reconstructed hGMPK SAXS models (GASBOR) overlaid on the crystal structure of the completely closed mGMPK conformation (*pdb* *1LVG (*[*Sekulic et al. 2002*](#_ENREF_9)*)*) and on the homology model of the completely closed hGMPK conformation.

| **hGMPK**  **SAXS model**  **(DAMMIN)** | **Conformation** | **NSD value mGMPK**  **(GMP + ADP)**  **(*pdb 1LVG)*** | **NSD value hGMPK**  **(homology model)** |
| --- | --- | --- | --- |
| **apo-form** | **open** | **1.2** | **1.2** |
| **GMP +**  **AMP-PNP** | **completely**  **closed 1** | **1.2** | **1.1** |
| **Ap5G** | **completely**  **closed 2** | **1.1** | **1.1** |
| **GMP + ADP** | **completely**  **closed 3** | **1.2** | **1.2** |

Table S4. Normalized spatial discrepancy (NSD) values for the reconstructed hGMPK SAXS models (DAMMIN) overlaid on the crystal structure of the completely closed mGMPK conformation (*pdb* *1LVG (*[*Sekulic et al. 2002*](#_ENREF_9)*)*) and on the homology model of the completely closed hGMPK conformation.

| **GMPK crystal structure**  **(pdb file)** | **Conformation** | **Bound ligands** | ***R_g_* (Å)**  **CRYSOL** |
| --- | --- | --- | --- |
| **1EX6 - A chain** | **yGMPK - Open** | **None** | **18.8** |
| **1EX7** | **yGMPK -**  **Partially closed** | **GMP** | **17.8** |
| **1LVG** | **mGMPK – Completely closed** | **GMP + ADP** | **17.2** |

Table S5. Sizes of known yeast ([Blaszczyk et al. 2001](#_ENREF_1)) and mouse GMPK crystal structures ([Sekulic et al. 2002](#_ENREF_9)).

**Supplementary references**

Blaszczyk J, Li Y, Yan H, Ji X (2001) Crystal structure of unligated guanylate kinase from yeast reveals GMP-induced conformational changes. Journal of molecular biology 307:247-257

Glatter O, Kratky O (1982) Small Angle X-ray Scattering. London: Academic Press

Guinier A, Fournet G (1955) Small Angle Scattering of X-rays. New York: Wiley

Hazra S, Ort S, Konrad M, Lavie A (2010) Structural and kinetic characterization of human deoxycytidine kinase variants able to phosphorylate 5-substituted deoxycytidine and thymidine analogues. Biochemistry 49:6784-6790

Kuhlendahl S, Spangenberg O, Konrad M, Kim E, Garner CC (1998) Functional analysis of the guanylate kinase-like domain in the synapse-associated protein SAP97. European journal of biochemistry / FEBS 252:305-313

Kumar V, Spangenberg O, Konrad M (2000) Cloning of the guanylate kinase homologues AGK-1 and AGK-2 from Arabidopsis thaliana and characterization of AGK-1. European journal of biochemistry / FEBS 267:606-615

Panavas T, Sanders C, Butt TR (2009) SUMO fusion technology for enhanced protein production in prokaryotic and eukaryotic expression systems. Methods in molecular biology (Clifton, NJ) 497:303-317

Prinz H, Lavie A, Scheidig AJ, Spangenberg O, Konrad M (1999) Binding of nucleotides to guanylate kinase, p21(ras), and nucleoside-diphosphate kinase studied by nano-electrospray mass spectrometry. The Journal of biological chemistry 274:35337-35342

Sekulic N, Shuvalova L, Spangenberg O, Konrad M, Lavie A (2002) Structural characterization of the closed conformation of mouse guanylate kinase. The Journal of biological chemistry 277:30236-30243

Svergun D (1992) Determination of the regularization parameter in indirect-transform methods using perceptual criteria. Journal of Applied Crystallography 25:495-503

Svergun D, Barberato C, Koch MHJ (1995) CRYSOL - a Program to Evaluate X-ray Solution Scattering of Biological Macromolecules from Atomic Coordinates. Journal of Applied Crystallography 28:768-773

Svergun DI, Petoukhov MV, Koch MH (2001) Determination of domain structure of proteins from X-ray solution scattering. Biophysical journal 80:2946-2953

Volkov VV, Svergun DI (2003) Uniqueness of ab initio shape determination in small-angle scattering. Journal of Applied Crystallography 36:860-864
